# Area vs. density: influence of visual variables and cardinality knowledge in early number comparison

**DOI:** 10.3389/fpsyg.2013.00805

**Published:** 2013-11-01

**Authors:** Roberto A. Abreu-Mendoza, Elia E. Soto-Alba, Natalia Arias-Trejo

**Affiliations:** Laboratorio de Psicolingüística, Facultad de Psicología, Universidad Nacional Autónoma de MéxicoMexico City, Mexico

**Keywords:** number comparison task, approximate number system, visual controls, cardinality knowledge, give a number task

## Abstract

Current research in the number development field has focused in individual differences regarding the acuity of children's approximate number system (ANS). The most common task to evaluate children's acuity is through non-symbolic numerical comparison. Efforts have been made to prevent children from using perceptual cues by controlling the visual properties of the stimuli (e.g., density, contour length, and area); nevertheless, researchers have used these visual controls interchangeably. Studies have also tried to understand the relation between children's cardinality knowledge and their performance in a number comparison task; divergent results may in fact be rooted in the use of different visual controls. The main goal of the present study is to explore how the usage of different visual controls (density, total filled area, and correlated and anti-correlated area) affects children's performance in a number comparison task, and its relationship to children's cardinality knowledge. For that purpose, 77 preschoolers participated in three tasks: (1) counting list elicitation to test whether children could recite the counting list up to ten, (2) give a number to evaluate children's cardinality knowledge, and (3) number comparison to evaluate their ability to compare two quantities. During this last task, children were asked to point at the set with more geometric figures when two sets were displayed on a screen. Children were exposed only to one of the three visual controls. Results showed that overall, children performed above chance in the number comparison task; nonetheless, density was the easiest control, while correlated and anti-correlated area was the most difficult in most cases. Only total filled area was sensitive to discriminate cardinal principal knowers from non-cardinal principal knowers. How this finding helps to explain conflicting evidence from previous research, and how the present outcome relates to children's number word knowledge is discussed.

## Introduction

Children encounter difficulties when learning the meaning of their first numerals and the cardinal principle—that the last word on a counting list equals the total number of items in a set (Wynn, 1990, 1992). Wynn (1992) reported that children go through a period of about 18 months between their ability to recite the counting list, at age two and a half, and their successful use of the list to know the cardinality of a set, around age four. Nonetheless, some children learn the meaning of their first numerals and the cardinal principle faster than others. In a meta-analysis of data obtained with the give-a-number task, in which children are tested to know what numbers they understand, Sarnecka and Lee (2009) found individual differences in children's understanding of numerals within a wide age range (30–55 months). For example, some 40-month-old children knew only the meaning of the numeral one, while others already knew the cardinal principle.

In recent years there has been a major interest in understanding the origins of these individual differences. Speaking to the importance of linguistic influences on numerical development, several authors have found relationships between children's performance in non-numerical tasks, such as noun and quantifier comprehension tasks, and their understanding of numerals and the cardinal principle (Barner et al., 2009; Negen and Sarnecka, 2011, 2012). For example, Negen and Sarnecka (2012) found a statistical correlation between children's comprehension of familiar nouns and the number of numerals they knew: children who knew more nouns were those who knew the meaning of more number words as well, even controlling for age. Similarly, Barner et al. (2009) found a relationship between children's understanding of quantifiers and their acquired numerals. This evidence supports the claim that language plays a critical role in children's understanding of the number concept (Barner et al., 2009; Carey, 2009).

Other authors, however, have emphasized the effect of core systems of number on later mathematical skills (Piazza, 2010). For example, it has been argued that the cardinal principle is acquired through a bootstrapping process with representations coming from the parallel individuation system, the system that allows us to represent small quantities based on one-to-one correspondence, and the Set-Based Quantificational System, which permits us to distinguish an individual from a set (Le Corre and Carey, 2007; Carey, 2009). A link between individual differences in mathematical skills and the approximate number system (hereafter ANS) acuity (Feigenson et al., 2013) has also been described. The ANS allows us to approximate the quantity of a set (Xu and Spelke, 2000). According to this proposal, ANS acuity is a predictor of children's and adults' performance in different mathematical tests. Typically, the acuity of the ANS is measured with a task in which participants have to decide which of two sets of items is larger; this ability is not dependent on the absolute difference between the quantities, but on the ratio between them (Feigenson et al., 2004). There has been a tendency to report ratio development in terms of trajectories: for example, newborns distinguish a ratio of 1:3 (Izard et al., 2009), and 3-year-olds distinguish ratios up to 3:4 and adults up to 9:10 (Halberda and Feigenson, 2008). The child's initial ability to compare quantities has been shown to be a predictor of her later ability across lifespan (Libertus and Brannon, 2010; Reeve et al., 2012).

An important prediction of the ANS linking proposal is that a person who can distinguish larger ratios would have a higher ANS acuity and would therefore have a better performance in mathematical abilities than someone who can only distinguish smaller ratios. Halberda et al. (2008) provided pioneering evidence to support this prediction, reporting that adults' individual differences in ANS acuity correlated with past performance in mathematical tests, even controlling for general intelligence and some cognitive abilities. Since then, several studies have further supported the link between individual differences in formal mathematics and ANS acuity (Libertus et al., 2011; Mazzocco et al., 2011b; Lourenco et al., 2012). Alternative evidence for this proposal comes from studies reporting that dyscalculic children have impaired ANS acuity (Piazza et al., 2010; Mazzocco et al., 2011a).

Importantly, several authors have suggested that the representations emerging from the ANS are independent of the stimulus modality (Dehaene and Changeux, 1993). Evidence to support this claim comes from a series of studies reporting that infants are able, based only on numerical information, to map visual stimuli with equivalent number auditory stimuli (Izard et al., 2009), as well as to distinguish number of sounds (Lipton and Spelke, 2003) and number of actions (Wood and Spelke, 2005). Adults can also, with little difficulty, make number comparisons across modalities, based on abstract representations of numbers. For example, Barth et al. (2003) showed that adults performed similarly when distinguishing two quantities across two different modalities (visual and auditory) or two quantities presented in only one modality.

Studies presenting visual stimuli have demonstrated that continuous variables (e.g., area or density) affect an infant's ability to discriminate quantities (see Mix et al., 2002). Several more recent studies have provided further evidence supporting the claim that infants as well as adults use not only number information but also visual cues in number comparison tasks (Gebuis and Reynvoet, 2012a,b,c). However, there are also habituation studies showing that young infants discriminate sets of objects more easily based on number information than visual information such as total filled area (Cordes and Brannon, 2009, 2011).

Although children's understanding of the meaning of numerals and the cardinal principle are major steps in number development (Carey, 2009), only a few studies have looked at the link between children's number word knowledge and their ANS acuity (Rousselle et al., 2004; Negen and Sarnecka, 2011; Wagner and Johnson, 2011). These studies have reached differing results. Wagner and Johnson (2011), using a modified version of the give-a-number task (Wynn, 1990, 1992) to test cardinality knowledge, found a correlation between children's understanding of numerals and their performance in a number discrimination task. Negen and Sarnecka (2011) did not encounter this correlation [see also, Rousselle and Noël (2008) for a similar result]. The divergent results of these two studies could be due to the different visual controls used in the number discrimination task: In the study by Wagner and Johnson (2011), dots were matched for total filled area (all quantities to be compared had the same total filled area), while Negen and Sarnecka (2011) used correlated and anti-correlated area stimuli (in half of the trials the image with the largest quantity of figures had the largest area, while in the other half, the image with the smallest quantity figures had the largest area). Rousselle et al. (2004) suggested that children's performance in number comparison tasks is affected by the type of visual control employed, which supports the idea that these divergent results could be due to the different visual controls employed; in this respect, area seems to be the most controlled continuous variable across studies, perhaps due to its importance in comparing quantities. Although Rousselle et al. (2004) demonstrated that the total filled area control increases the difficulty in children's discrimination between two quantities as compared with visual controls like density and contour length, these authors did not use the correlated and anti-correlated area control typically used in studies with children (Halberda and Feigenson, 2008; Mazzocco et al., 2011b; Negen and Sarnecka, 2011). Thus, it remains unknown whether correlated and anti-correlated area control makes children's number discrimination easier or more difficult than with other visual controls. Another unresolved issue is how different visual controls relate to number word knowledge. Although the studies by Wagner and Johnson (2011) and Negen and Sarnecka (2012) have provided some insights, this issue has not yet been directly explored. The only way to eliminate reliance on all possible sources of visual information would be a study in which all controls are taken into account, which remains a task for future study. Nevertheless, our work contributes to clarify a few aspects of this enigma.

The two questions raised here—how children's ability to compare quantities is influenced by visual cues, and how the use of different visual controls relates to their knowledge of number words—have important theoretical and methodological implications. Theoretically, if children's performance were affected by the different visual controls used, that would question whether children are really extracting number information to compare quantities or are using multiple visual cues to make decisions about number, as recently suggested by Gebuis and Reynvoet (2012b). Moreover, if children's ability to distinguish quantities is highly influenced by the specific visual information presented, that would suggest the existence of a more general skill based on continuous variables, one which might form the basis of number word knowledge. Methodologically speaking, we might be able to determine which is the most effective visual control for the evaluation of early quantity discrimination.

The current study aims to explore these issues in the study of children's numerical abilities with two tasks: the give-a-number task (Wynn, 1990, 1992) as an assessment of children's number word knowledge, and a number comparison task using different visual controls (density, total filled area, and correlated and anti-correlated area). Area information is a commonly used control to prevent children from using visual properties of the stimuli to compare quantities, either by controlling the total area or by using it in a misleading way. Density was used to compare alternative visual information with area information.

## Materials and methods

### Participants

Seventy-seven preschool children (mean age 4;1; range 3;2 to 5;1, 41 females) participated in the experiment. Three additional children took part but their data were excluded due to lack of participation in either the give-a-number task (*n* = 1) or the number comparison task (*n* = 2). Children were recruited and evaluated in local preschools in Mexico City, Mexico.

Children were tested individually in a quiet space such as the school library or a classroom. Prior to the tasks of interest, children were always tested first on the Count List Elicitation task with the aim of confirming that they could recite the counting list. After this task, the give-a-number task and then the number comparison task were performed. Children were assigned in a pseudo-counterbalanced manner with respect to one of the three visual control conditions of the number comparison task (density, total filled area, and correlated and anti-correlated area) to make sure that CP-knowers and non-CP-knowers were assigned equally to each visual control.

### Count list elicitation task

Ten colored plastic turtles each measuring 2.2 × 1.0 inches were employed.

### Give-a-number (GN) task

Thirteen turtles each measuring 2.2 × 1.0 inches as well as a plastic container were used.

### Number comparison task

#### Visual stimuli

In order to explore how different visual controls affect children's performance in number comparison tasks, three different control conditions were created: density, total filled area, and correlated and anti-correlated area. Each child saw only one condition. The three controls employed are explained below.

#### Density

All figures were the same size, and density was controlled by keeping the same space between the stimuli (see Figure 1.1).

**Figure 1:**
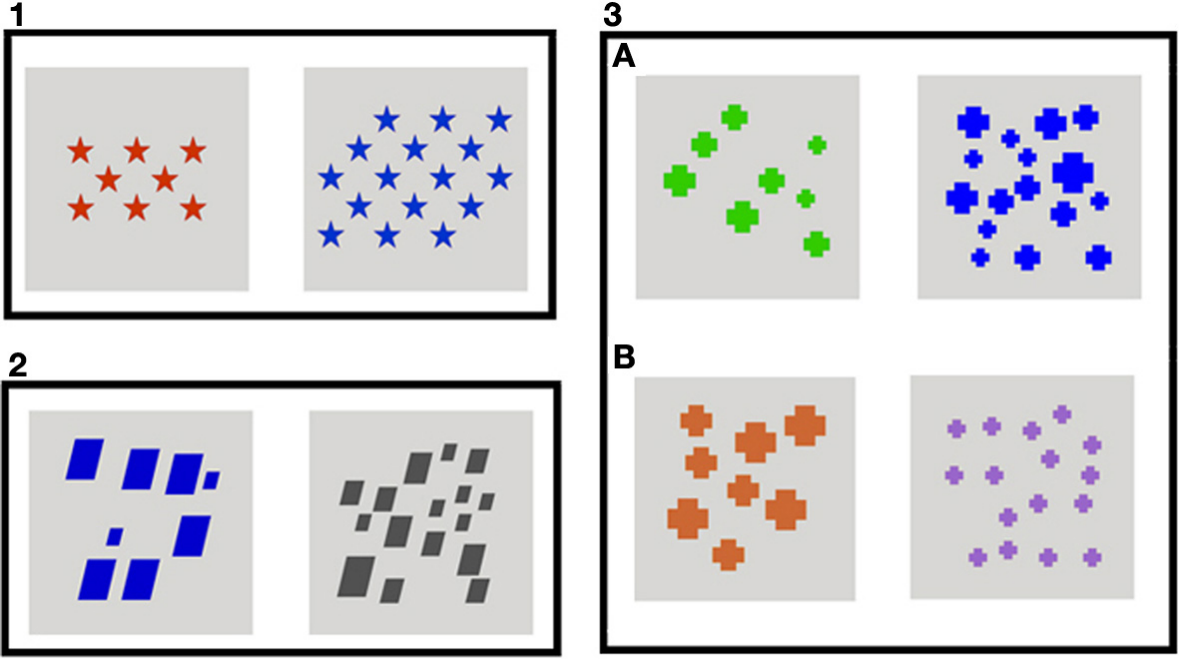
**Example: Comparison 8 vs. 16 across all different visual controls. (1)** Density, **(2)** Total Filled Area, **(3A)** Correlated Area, and **(3B)** Anti-correlated Area trials.

#### Total filled area

Four different sizes of figures were created to prevent children from responding based on the change of size. In all comparisons, the two quantities to be compared had the same total filled area; for example, in the comparison 8 vs. 16, the image with 8 triangles had 30,638 pixels of total filled area, and the image with 16 triangles had the same number of pixels. Figures were distributed randomly on an invisible grid of 12 × 12 cm. The 16 × 16 cm square was not employed in its total area to avoid presenting visual stimuli close to the corners (see Figure 1.2).

#### Correlated and anti-correlated area

As with the total filled area control stimuli, four different sizes of figures were created. In the Correlated Area trials, the image with the largest quantity of figures had the largest area; for example, in the correlated version of the 8 vs. 16 comparison, the image with 8 figures had 16,448 pixels of total filled area, while the image with 16 figures had 32,896 pixels (see Figure 1.3A). However, in the Anti-correlated Area trials, the image with the smallest quantity of figures had the largest area; for example, in the anti-correlated version of the 8 vs. 16 comparison, the image with 8 figures had 32,896 and the image with 16 figures had 16 448 pixels (see Figure 1.3B). Moreover, images had the same ratio in number as in area in both types of trials, correlated and anti-correlated; for example, 8 and 16 and 16,448 and 32,896 pixels both represent a ratio of 1:2. Figures were also distributed on a grid of 12 × 12 cm.

### Design and procedure

#### Count list elicitation task

Children were presented with a single row of 10 colored plastic turtles and were asked in Spanish to count them. If initially a child didn't want to cooperate, the experimenter encouraged her by helping with the first number word and by pointing at one turtle per elicited number word.

#### Give-a-number (GN) task

The *GN* task (Wynn, 1990, 1992) lasted approximately 5 min and was also conducted in Spanish. The child was seated in a chair by herself in front of the Experimenter (E). The child was told that the experimenter (E) wanted to play a game with turtles. E placed the plastic container with 13 turtles in front of the child and said “*Mira estas tortugas*, *son bonitas*, ¿*te gustan*? ¿*quieres jugar con ellas*?” (Look at these turtles, they are nice, do you like them? Do you want to play with them?) Once the child said “*Sí*” (Yes), E asked “¿*Podrías darme una tortuga*? ¿*Podrías poner una tortuga en la mesa*?” (Could you give me one turtle? Could you put one turtle on the table?)” In the absence of a response, E repeated the same two questions a maximum of two additional times. If the child gave one turtle, E provided positive feedback and proceeded to ask for two: “¿*Podrías darme dos tortugas*? ¿*Podrías poner dos tortugas en la mesa*?” (Could you give me two turtles? Could you put two turtles on the table?) Once the child placed a number of turtles on the table E asked “¿*Puedes contarlas para asegurar que hay dos*?” (Can you count them to make sure there are two?) If the child gave the correct number of turtles and counted correctly, E proceeded to ask for the next number, up to six turtles. If the child did not provide the correct number and/or did not count correctly, E said “*Pero yo quiero dos tortugas*, ¿*puedes arreglarlo para que haya dos*?” (But I want two turtles, can you fix it so that there are two?) and waited to see if the child changed her response. If the child did not fix her response, E went back to the preceding number. If the child succeeded once and failed once on the same number, E asked a third time for the target number; if the child succeeded again, E proceeded to ask for the next number. The task stopped either when the child failed to give the same number twice or when she gave six turtles correctly two times. Children were categorized as knowers of a given number when they succeeded in two of three trials for the same number. Classification of children's cardinality knowledge was modeled on Le Corre and Carey (2007): if a child succeeded at all the requested numbers (up to six turtles) that child was categorized as a cardinal principle knower (CP-knower), while a child who knew fewer than six was categorized as a non-cardinal principle knower (non-CP-knower).

#### Number comparison task

This task was an adapted version of Halberda and Feigenson (2008). SuperLab software was used to administrate the presentation of the trials. The study, also conducted in Spanish, started by inviting the child to play a computer game. The child was seated in a chair by herself, approximately 30 cm from the screen (viewing area 41 × 23 cm). The experimenter explained to the child that she was going to see figures on both sides of the screen and that she had to touch or point to the side with more figures.

The task consisted of 24 trials. In each trial, children had to compare two different sets of one of six geometric figures employed: squares, circles, crosses, stars, triangles and parallelograms (e.g., 8 triangles vs. 16 triangles). Different figures were used in order to maintain children's interest in the task. The figures were placed on a square gray background (16 × 16 cm). Three ratios (1:2, 2:3, and 3:4) and two different sizes of quantities (small and large) were used, resulting in six different comparisons: 1 vs. 2, 2 vs. 3, 3 vs. 4, 8 vs. 16, 10 vs. 15, and 9 vs. 12. For each comparison, two different visual arrangements were created, resulting in 12 different pairs of images, which were each presented the same number of times during the task. Each quantity was also presented the same number of times on the right and left sides of the screen. The task was divided into a small quantity block (1 vs. 2, 2 vs. 3, 3 vs. 4) and a large quantity block (8 vs. 16, 10 vs. 15, and 9 vs. 12). Half of the participants were exposed to the small quantity block first; the others saw the large quantity block first.

The task started with two practice trials in which a set of figures appeared on a given side of the screen accompanied by the phrase “¡*Ve*!” (See!). After 2000 ms, the set disappeared and a different set of figures appeared on the other side, also for 2000 ms, accompanied by the phrase “!*Mira*!” (Look!). After the 4000 ms, a blank screen was presented for 1300 ms while the instruction “¿*Dónde hay más*?” (Where are there more?) was heard through the computer speakers. Finally, both sets of figures were displayed simultaneously until the child responded by either touching or pointing to a set of figures. Feedback was given in both practice trials: If children answered incorrectly, a sad red face appeared and the trial was repeated; if the answer was correct, a smiling green face appeared and they could continue to the next trial. Practice trials presented comparisons that were never presented in test trials. Test trials were identical to practice trials, except that the duration of each set lasted only 2500 ms and children did not receive feedback.

## Results

### Count list elicitation task

Only 10 of the 80 children participating in this task were unable to count to ten. Similar results have been obtained elsewhere (Le Corre and Carey, 2007).

### GN task

Forty children were categorized as non-CP-knowers, and the other 36 as CP-knowers; one did not know the meaning of any number and was therefore eliminated from the sample.

Children were assigned to one of the three different visual controls in a pseudo-counterbalanced manner, resulting in the following distribution: density control, 15 non-CP-knowers and 11 CP-knowers; total filled area control, 13 non-CP-knowers and 12 CP-knowers; correlated and anti-correlated control, 12 non-CP-knowers and 13 CP-knowers.

### Number comparison task

#### Coding

Children's correct responses were coded as 1, while incorrect responses were coded as −1; the chance level score was thus 0.

#### Item analysis

An item analysis was performed to determine whether there was a trial type whose mean response was more than two standard deviations from that of all the different trial types. There were a total of 72 trial types: 6 ratio comparisons × 3 control conditions × 2 visual arrangements × 2 target sides. Mean scores in one of the two 3:4 ratio arrangements with small quantities in the density control were two standard deviations below the mean scores for all other trial types in that control. This trial type (2.8% of the originally presented trials) was therefore eliminated from further analysis.

#### Preliminary analysis

A grand five-way repeated measures ANOVA was performed with size of quantities (Small and Large) and ratio (1:2, 2:3, and 3:4) as within-subject factors, and visual control (density, total filled area, and correlated and anti-correlated area visual control), cardinality knowledge (non-CP-knowers and CP-knowers), and Order of Presentation of the size of quantities (Large-Small vs. Small-Large) as between-subject factors, revealed main effects for Ratio [*F*_(2, 71)_ = 12.18, *p* = 0.000, η^2^ = 0.17], visual control [*F*_(2, 71)_ = 20.74, *p* = 0.000, η^2^ = 0.40], and cardinality knowledge [*F*_(1, 72)_ = 24.30, *p* = 0.000, η^2^ = 0.28]. There were no significant effects for order of presentation of the size of quantities, or for size of quantities itself. Therefore, further analyses did not consider these two factors.

#### Main analysis

**Figure 2:**
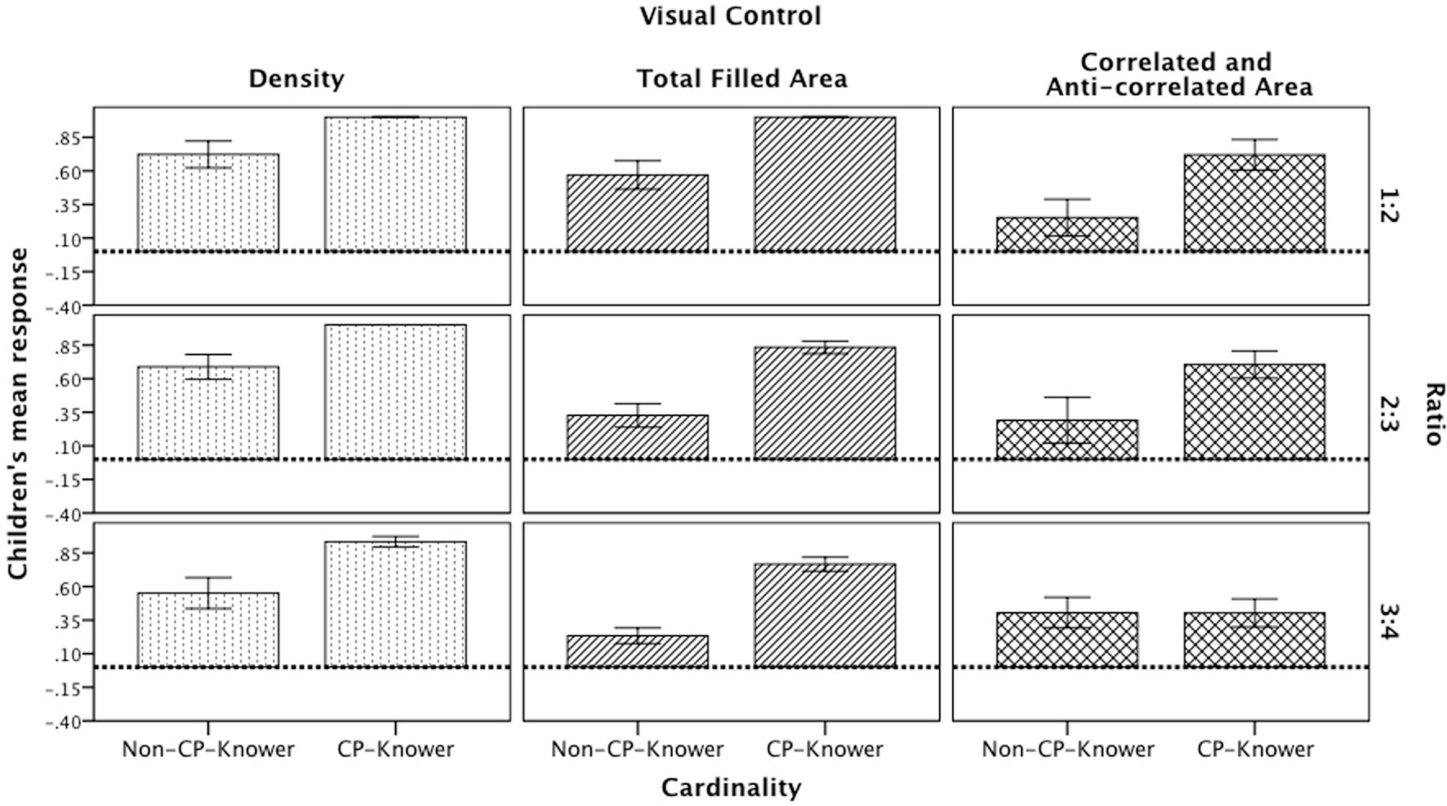
**Children's mean responses divided by visual control, ratio, and cardinality knowledge.** Dotted line represents chance level (0).

A three-way repeated measures ANOVA with Ratio (1:2, 2:3, and 3:4) as a within-subject factor, and visual control (density, total filled area, and correlated and anti-correlated visual control) and cardinality knowledge (non-CP-knowers vs. CP-knowers) as between-subjects factors revealed main effects for ratio [*F*_(2, 74)_ = 8.96, *p* = 0.000, η^2^ = 0.11], Visual Control [*F*_(2, 74)_ = 10.2, *p* = 0.000, η^2^ = 0.22] and cardinality knowledge [*F*_(1, 75)_ = 32.691, *p* = 0.000, η^2^ = 0.09]. Also, a three-way interaction between ratio, visual control, and cardinality knowledge [*F*_(4, 72)_ = 3.52, *p* = 0.009, η^2^ = 0.09] was encountered (see Figure 2). In the following sections we describe these results in detail.

#### Ratio effect

Children's performance was above chance (chance level score = 0) in the three ratios. However, there were some variations: The 3:4 ratio (*M* = 0.54; *SD* = 0.41) was more difficult than 1:2 [*M* = 0.63; *SD* = 0.42; *t*_(75)_ = 3.95, *p* < 0.001] and 2:3 [*M* = 0.70; *SD* = 0.41; *t*_(75)_ = 2.45, *p* < 0.05], although there were no differences between the 1:2 and 2:3 ratios. Importantly, there were more specific significant results from the 3-way interaction.

***Density control***. *Post-hoc* analyses revealed that non-CP-knowers and CP-knowers performed similarly across the three ratios in this control.

***Total filled area control***. There was a significantly different performance between the 1:2 (*M* = 0.57; *SD* = 0.38) and 2:3 ratios (*M* = 0.33; *SD* = 0.32); the scores were also different between the 1:2 and 3:4 ratios (*M* = 0.23; *SD* = 0.21). Thus, non-CP-knowers were better in trials presenting a 1:2 ratio than in those of the other two ratios. The same pattern of results was encountered with the cardinal-principle knowers (1:2 ratio *M* = 1, *SD* = 0 > 2:3 ratio *M* = 0.83, *SD* = 0.16 ≈ 3:4 *M* = 0.77, *SD* = 0.18).

***Correlated and anti-correlated control***. Only CP-Knowers were better at comparing quantities involving a 1:2 (*M* = 0.72; *SD* = 0.41) and a 2:3 ratio (*M* = 0.70; *SD* = 0.36) than a 3:4 ratio (*M* = 0.40; *SD* = 0.37). Table 1 presents the statistical values for all comparisons discussed here.

**Table 1:** **Statistics for the ratio effect divided by visual control and cardinality knowledge**.

**Ratio effect**			
	**(d.f.)**	***t***	***p*-value**
**DENSITY**			
*Non-CP-Knowers*			
1:2 vs. 2:3	14	0.59	0.56
1:2 vs. 3:4	14	1.85	0.085
2:3 vs. 3:4	14	1.49	0.16
*CP-Knowers*			
1:2 vs. 2:3	–	–	–
1:2 vs. 3:4	10	1.66	0.13
2:3 vs. 3:4	10	1.66	0.13
**TOTAL FILLED AREA**			
*Non-CP-Knowers*			
1:2 vs. 2:3	12	2.79	0.02
1:2 vs. 3:4	12	2.78	0.02
2:3 vs. 3:4	12	0.99	0.34
*CP-Knowers*			
1:2 vs. 2:3	11	3.55	0.005
1:2 vs. 3:4	11	4.54	0.001
2:3 vs. 3:4	11	0.98	0.33
**CORRELATED AND ANTI-CORRELATED AREA**			
*Non-CP-Knowers*			
1:2 vs. 2:3	11	0.22	0.83
1:2 vs. 3:4	11	1.23	0.25
2:3 vs. 3:4	11	0.98	0.35
*CP-Knowers*			
1:2 vs. 2:3	12	0.18	0.86
1:2 vs. 3:4	12	3.21	0.007
2:3 vs. 3:4	12	2.67	0.02

#### Visual control effect

Overall, among the three visual controls manipulated, density was the easiest visual control for discrimination of quantities, as compared with the total filled area [*t*_(49)_ = 2.08, *p* < 0.05] and the correlated and anti-correlated control [*t*_(49)_ = 3.28, *p* < 0.01]. However, there were no differences between the total filled area control and the correlated and anti-correlated [*t*_(48)_ = 1.46, *p* = n.s.]. We describe the 3-way interaction:

***1:2 ratio***. analyses for the non-CP-knowers revealed a significant difference between density control (*M* = 0.72; *SD* = 0.38) and correlated and anti-correlated control (*M* = 0.25; *SD* = 0.47). For this same ratio, CP-knowers also had a significant difference between density control (*M* = 1; *SD* = 0) and correlated and anti-correlated control (*M* = 0.72; *SD* = 0.41), and a significant difference between total filled area control (*M* = 1; *SD* = 0) and correlated and anti-correlated control.

***2:3 ratio***. Non-CP-knowers' performance was significantly better in the density control (*M* = 0.69; *SD* = 0.36) than in the total filled area control (*M* = 0.33; *SD* = 0.32); they were also significantly better in the density control than in the correlated and anti-correlated control (*M* = 0.29; *SD* = 0.59). CP-knowers showed the same patterns of results (density control *M* = 1, *SD* = 0 > total filled area *M* = 0.83, *SD* = 0.16 ≈ correlated and anti-correlated *M* = 0.70, *SD* = 0.36).

***3:4 ratio***. Non-CP-knowers performed significantly differently between density control (*M* = 0.55; *SD* = 0.45) and total filled area control (*M* = 0.23; *SD* = 0.21). Although CP-knowers showed the same response pattern than non-CP-knowers in the 3:4 ratio, they also showed a significantly different performance between density control (*M* = 0.93; *SD* = 0.13) and correlated and anti-correlated control (*M* = 0.40; *SD* = 0.37), as well as a significant difference between total filled area control (*M* =0.77; *SD* = 0.18) and correlated and anti-correlated control. See Table 2 for detailed statistics.

**Table 2:** **Statistics for the visual control effect divided by ratio and cardinality knowledge**.

**Visual control effect**			
	**(d.f.)**	***t***	***p*-value**
**RATIO 1:2**			
*Non-CP-Knowers*			
Density vs. total filled area	26	1.05	0.3
Density vs. correlated and anti-correlated area	25	2.85	0.009
Total filled area vs. correlated and anti-correlated area	23	1.86	0.075
*CP-Knowers*			
Density vs. total filled area	–	–	–
Density vs. correlated and anti-correlated area	22	2.26	0.034
Total filled area vs. correlated and anti-correlated area	23	2.37	0.027
**RATIO 2:3**			
*Non-CP-Knowers*			
Density vs. total filled area	21	3.39	0.003
Density vs. correlated and anti-correlated area	25	2.17	0.039
Total filled area vs. correlated and anti-correlated area	23	0.19	0.85
*CP-Knowers*			
Density vs. total filled area	21	3.39	0.003
Density vs. correlated and anti-correlated area	22	2.66	0.014
Total filled area vs. correlated and anti-correlated area	23	1.11	0.277
**Ratio 3:4**			
*Non-CP-Knowers*			
Density vs. total filled area	26	2.34	0.027
Density vs. correlated and anti-correlated area	25	0.88	0.39
Total filled area vs. correlated and anti-correlated area	23	1.38	0.18
*CP-Knowers*			
Density vs. total filled area	21	2.52	0.02
Density vs. correlated and anti-correlated area	22	4.43	0.000
Total filled area vs. correlated and anti-correlated area	23	3.038	0.006

***Cardinality knowledge effect***. In general, CP-knowers were better at discriminating quantities than non-CP-knowers across all ratios and all visual controls [*t*_(74)_ = 4.79, *p* < 0.001]. Nonetheless, closer analysis revealed that this was not true for the 3:4 ratio in the correlated and anti-correlated control, in which there were no differences between groups of children. See Table 3 for detailed statistics.

**Table 3:** **Statistics for the cardinality knowledge effect divided by visual control and ratio**.

**Cardinality knowledge effect**			
**(Non-CP-Knowers vs. CP-Knowers)**			
	**(d.f.)**	***t***	***p*-value**
**DENSITY**			
Ratio 1:2	24	2.38	0.026
Ratio 2:3	24	2.88	0.008
Ratio 3:4	24	2.73	0.012
**TOTAL FILLED AREA**			
Ratio 1:2	23	3.93	0.001
Ratio 2:3	23	4.94	0.000
Ratio 3:4	23	6.84	0.000
**CORRELATED AND ANTI-CORRELATED AREA**			
Ratio 1:2	23	2.65	0.014
Ratio 2:3	23	2.14	0.043
Ratio 3:4	23	0.012	0.99

To further explore the effect of cardinality knowledge in children's ability to compare two sets of quantities, and rule out the possibility that this could simply be an age effect, we performed a linear regression analysis on children's response scores in each visual control condition and for each ratio, using age and cardinality knowledge. We found that for children's performance in the density control, age was the only significant predictor when they saw comparisons differing by a 2:3 ratio (β = 0.56; *p* = 0.004). Older children were better than younger children at this ratio; however, neither age nor cardinality knowledge were significant predictors in the 1:2 and 3:4 ratio. In the total filled area control, cardinality knowledge was the only significant predictor when children saw comparisons involving the 2:3 (β = 0.62; *p* = 0.005) and 3:4 (β = 0.73; *p* < 0.001) ratios, while in the 1:2 ratio, cardinality knowledge was marginally significant (β = 0.44; *p* = 0.051): CP-knowers are better at discriminating two quantities involving 2:3 and 3:4 ratios than non-CP-knowers. Lastly, in the correlated and anti-correlated area control, Age was the only significant predictor found in the 1:2 ratio (β = 0.60; *p* = 0.001): Older children had higher mean responses than younger ones. No other significant predictor was found for the 2:3 and 3:4 ratios in the correlated and anti-correlated area control. While cardinality knowledge seems to have an effect on children's ability to discriminate two quantities in the total filled area control condition in all ratios, age has an effect only on density control for the 2:3 ratio, and correlated and anti-correlated control for the 1:2 ratio. If neither cardinality knowledge nor age are strong predictors for children's performance in most ratios for density and correlated and anti-correlated controls, then an alternative factor may explain our results.

***Correlated vs. anti-correlated trials***. Although some studies report no differences between performance in the correlated and anti-correlated trials (Halberda and Feigenson, 2008), there are studies reporting opposite results (Hurewitz et al., 2006). Thus, we decided to explore whether children were equally accurate in these two types of trials. A repeated measures ANOVA with type of trial (correlated vs. anti-correlated) as a within-subject factor did not yield a significant effect [*F*_(1, 24)_ = 3.94, *p* = 0.059, η^2^ = 0.14]. Although children had higher scores in the correlated trials (*M* = 0.60; *SD* = 0.45) than in the anti-correlated ones (*M* = 0.43; *SD* = 0.42), this difference was not significant.

## Discussion

Despite the fact that children can clearly discriminate numerosities from a very early stage of development (Xu and Spelke, 2000; Turati et al., 2013), and even though newborn infants can associate visual-spatial arrays with auditory sequences on the basis of number (Izard et al., 2009), there is evidence showing that children understand the cardinal principle at very different ages between three and four (Wynn, 1990, 1992; Sarnecka and Lee, 2009). Researchers have proposed various explanations regarding these individual differences. One of the most widespread views is the one that proposes the existence of a relationship between the ANS and mathematical knowledge (Feigenson et al., 2013). Nonetheless, it has also been argued that the influence of diverse visual cues could affect the acuity of ANS representations and the relationship between mathematical skills and the ANS (Gebuis and Reynvoet, 2012c; Gilmore et al., 2013). Therefore, this study aimed not only to determine the influence of different visual controls in children's performance in a number comparison task, but also to identify to what degree this influence was the result of children's cardinality knowledge.

As previously mentioned, Rousselle et al. (2004) found that total filled area, but not density, contour length, or heterogeneous size, was a significant control for differentiating children who knew some number from children who did not know any number. Other researchers have reported that correlated and anti-correlated area control trials also correlate with mathematical skills (e.g., Halberda et al., 2008). Therefore, we aimed to test three different visual controls—density, total filled area, and correlated and anti-correlated area—to clarify the role that different visual cues play in early discrimination of quantities. We also tested how children's ability to discriminate between two quantities within the different visual controls introduced correlates with mastery of cardinality knowledge.

In order to accomplish these objectives, we performed a number comparison task in which preschool children were shown pairs of images and asked to touch/point at the image that had a larger quantity of items. Three different ratios (1:2, 2:3, and 3:4), two different sizes of quantities (small and large) and three different visual controls (density, total filled area, and correlated and anti-correlated area) were manipulated. Children were divided into non-CP-knowers and CP-knowers according to the give-a-number task (Wynn, 1990, 1992). We discuss the outcome of this research giving the effects of ratio, visual control, and cardinality knowledge.

However, before explaining these significant effects, one non-significant effect worth mentioning is the lack of difference in children's performance between the blocks of large and small quantities. There is a debate as to whether large and small quantities are processed by the same system. The results of the present study suggest that children used only the ANS, regardless of the size of the quantity. If so, a possible explanation of why children did not use the parallel individuation system, the system that has been posited for the processing of small quantities, could be that the visual arrangement of the stimuli within a given set was not appropriate to evoke it. A study by Hyde and Wood (2011), reporting that the proximity of stimuli determines which system is used, supports this conclusion.

### Ratio effect

Our results showed that children performed above chance in all ratio comparisons (selection of the image with more objects), even in the most difficult case (3:4 ratio), which is consistent with previous findings (Halberda and Feigenson, 2008). Nevertheless, children's performance was different according to the visual control they saw. In the total filled area control trials, children's performance was affected by the ratio difference: As the ratio became smaller, it also became more difficult to compare quantities. A ratio effect is one of the main characteristics of the ANS (Feigenson et al., 2004; Piazza, 2010). Thus, these results could be taken as evidence that children use the ANS to compare quantities when they are exposed to the total filled area control, regardless of their cardinality knowledge. However, the outcome also suggests that only CP-knowers are also using ANS to compare quantities in the correlated and anti-correlated area control trials. In contrast, in density control trials neither CP-knowers nor non-CP-knowers use numerical cues: they perform similarly in all ratios. Thus, children may be extracting information from other visual cues to perform the density control trials.

### Visual control effect

Children's performance in the number comparison task is influenced by the visual control introduced. In most of the cases, density control was the easiest visual control and correlated and anti-correlated area the most difficult. The ease of the density control comes as no surprise; a previous study (Rousselle et al., 2004) also reported that density control was easier than total filled area control. A striking result is the difference encountered between children's performance in the total filled area trials and the correlated and anti-correlated area trials. These two controls have been used interchangeably in previous studies assessing early numerical discrimination, but why is it more difficult to process correlated and anti-correlated area than total filled area in our numerical comparison task? A possible explanation is that children perform different processes in the two different visual controls. In the total filled area control, children can directly compare the two quantities and identify the set with more objects. However, in the correlated and anti-correlated area control, children might need to perform multiple processes: inhibit the misleading information of total surface area in the anti-correlated trials and identify which image has more objects. They may also need to change strategies when they compare items in correlated trials as opposed to anti-correlated ones. The interference of the area and number information in the correlated and anti-correlated area control have led some researchers to call this visual control a “Stroop-like manipulation” (Iuculano et al., 2008). Therefore, the difficulty of processing correlated and anti-correlated areas might be due to the requirement of performing some executive functions in addition to extracting number information.

Previous research using correlated and anti-correlated area control has found a relationship between children's ability to perform a numerical comparison task and their mathematical achievement (Libertus et al., 2011). In addition, Simms et al. (2013) found a relationship between children's mathematical reasoning and their executive functions such as cognitive flexibility. Thus, it may be possible that a relationship exists between children's performance in a numerical comparison task, using correlated and anti-correlated area control, and their executive functions. This possibility has been previously tested by Rousselle and Noël (2008), who failed to find a correlation between children's performance in a numerical comparison task and the day-night task (a task that tests children's inhibition). Another kind of task specifically designed to assess switching of cognitive strategies related to perceptual variables is needed. The relationship of these tasks to children's performance in a correlated and anti-correlated area control could explain more specifically the ability to choose numerosity over area in this control. In such a task, children's success in the Anti-correlated trials would demonstrate their use of numerosity to solve the task; success in Correlated area trials would demonstrate their use of Area and Number information.

### Cardinality knowledge effect

The results of this study show that overall, CP-knowers are better at comparing quantities than non-CP-knowers. However, outperformance of CP-knowers was more evident in the total filled area across the three ratios. This result cannot be explained by children's age: this factor was not a consistent predictor of children's performance. A plausible explanation for this outcome might be due to factors involved in becoming a CP-knower, for example the suggestion by Rousselle et al. (2004) that acquiring cardinality knowledge may provide children with a greater sense of “discreteness.”

This outcome may also help us to understand divergent results in the literature. Wagner and Johnson (2011) found a correlation between children's number word knowledge and their performance in a numerical comparison task using total filled area control. Negen and Sarnecka (2011), however, did not find such a relationship using correlated and anti-correlated area control. The divergent results between these two studies, even where both controlled for age, may be due to the fact, suggested by our results, that children's performance in total filled area control is a better predictor for cardinality knowledge than their performance in the correlated and anti-correlated control. The fact that executive and visual-spatial skills could be needed for mathematical learning (Raghubar et al., 2010) and that these same skills may also be needed to perform accurately in a numerical comparison task using correlated and anti-correlated area may explain why Libertus et al. (2011), using this area control, found a correlation between children's performance in a numerical task and their mathematical achievement. Children's executive functions are likely to contribute to the correlation reported between mathematical skills and performance in a number comparison task.

### Correlated and anti-correlated trials

Children's accuracy in the correlated trials was not significantly different from their accuracy in Anti-correlated trials, consistent with the findings of Halberda and Feigenson (2008) but not those of Hurewitz et al. (2006). Despite the absence of a significant difference, the correlated and anti-correlated control was still the most difficult for children, suggesting that their performance was susceptible to the misleading area information in Anti-correlated trials. Future studies should test this assumption further by exposing children to correlated and anti-correlated trials separately.

In summary, the outcome of the current research demonstrates that children's performance in a number comparison task is highly influenced by the visual properties of the stimuli. Researchers have suggested that the correlated and anti-correlated control taps into children's representation of the ANS, given that this control prevents children from using any visual cues (Halberda and Feigenson, 2008). However, according to our data, their performance in this control did not correlate with cardinality knowledge. In contrast, we found that the total filled area control was the only visual control that correlated with cardinality knowledge when controlling for Age. Two explanations could be given for this outcome: (1) Children might be using number information only in the total filled area control, which would explain the existence of the correlation in their performance in this condition; or (2) Children use item size in the total filled area control, suggesting that they use continuous variables to solve number comparison tasks and that the ability to compare continuous magnitudes (e.g., item size) is the one related to cardinality knowledge. Item size is the visual cue that is not manipulated in the total filled area control, since the set with the larger quantity was also the one with the greater number of smaller items.

The present study has several limitations: First, instead of dividing children by their number knowledge level, we divided children by their cardinality principle knowledge; thus, it can only be inferred that dividing children into more fine-grained category, such as children's CP-knower level, would produce the same results. Another limitation is that the current study only employed three visual controls out of the several reported in the literature; further studies should investigate, for example, how proximity of the figures within a given set affects children's performance. The duration of our trials could also be taken as a limitation; children could have counted in the small size quantities block, although there is evidence that suggests that non- and CP-knowers do not use counting to determine the cardinality of a small set (Le Corre et al., 2006). Additionally, we did not find differences between children's performance in the small and large quantities blocks, which implies that they did not use different strategies for different quantity sizes.

Although the kind of information and strategies children use in the visual controls employed in this study has not been fully resolved, we have contributed to an understanding of the relationship between cardinality knowledge and ANS acuity, and how this relationship is affected by the visual properties of stimuli in the number comparison task. Future studies should investigate whether children are using number information alone, or if they need to include other strategies, such as executive functions, to compare quantities. It should also explore how individual visual controls have distinct impacts on different mathematical abilities.

### Conflict of interest statement

The authors declare that the research was conducted in the absence of any commercial or financial relationships that could be construed as a potential conflict of interest.
